# Correlation of Low Levels of *α*-1 Antitrypsin and Elevation of Neutrophil to Lymphocyte Ratio with Higher Mortality in Severe COVID-19 Patients

**DOI:** 10.1155/2021/5555619

**Published:** 2021-04-28

**Authors:** Ghazaleh Shimi, Golbon Sohrab, Katayoun Pourvali, Arman Ghorbani, Farinaz Hosseini Balam, Khalil Rostami, Hamid Zand

**Affiliations:** ^1^Department of Cellular and Molecular Nutrition, Faculty of Nutrition and Food Technology, National Nutrition and Food Technology, Research Institute, Shahid Beheshti University of Medical Sciences, Tehran, Iran; ^2^Department of Clinical Nutrition and Dietetics, Faculty of Nutrition and Food Technology, National Nutrition and Food Technology, Research Institute, Shahid Beheshti University of Medical Sciences, Tehran, Iran; ^3^Student Research Committee, Department of Cellular and Molecular Nutrition, Faculty of Nutrition Sciences and Food Technology, Iran; ^4^Department of Plastic and Reconstructive Surgery, Shahid Modares Educational Hospital, Shahid Beheshti University of Medical Sciences, Tehran, Iran

## Abstract

**Background:**

Variations in COVID-19 prevalence, severity, and mortality rate remain ambiguous. Genetic or individual differences in immune response may be an explanation. Moreover, hyperinflammation and dysregulated immune response are involved in the etiology of severe forms of COVID-19. Therefore, the aim of the present study was to analyze serum alpha-1 antitrypsin (AAT) levels, as an acute-phase plasma protein with immunomodulatory effect and neutrophil to lymphocyte ratio (NLR) as a marker of inflammation response in severe COVID-19 illness.

**Methods:**

In this retrospective observational cohort study, 64 polymerase chain reaction (PCR) positive COVID-19 hospitalized patients were studied for AAT, C-reactive protein (CRP), erythrocyte sedimentation rate (ESR), troponin, complete blood count (CBC), random blood sugar, serum glutamate oxaloacetate transaminase (SGOT), serum glutamate pyruvate transaminase (SGPT), and arterial oxygen saturation (O2sat) at admission and during hospitalization.

**Results:**

The results showed that hospitalized patients with COVID-19 had low serum levels of AAT and high CRP levels at the first days of hospitalization. In particular, the percentages of individuals with low, normal, and high AAT levels were 7.80%, 82.80%, and 9.40%, respectively, while high and low values of CRP accounted for 86.70% and 13.30% of patients. Most of the patients had an upward neutrophil to lymphocyte ratio (NLR) trend, with a higher mortality rate (*p* < 0.05) and troponin levels (*p* < 0.05). However, comorbidities, CRP alterations, ESR alterations, nonfasting blood sugar, SGOT, SGPT, O2sat, RBC, and PLT values were not significantly different between the NLR downward and upward trend groups.

**Conclusions:**

The current study revealed that severe COVID-19 patients had low serum AAT levels related to CRP values. Therefore, AAT response may be considered as a new mechanism by which some COVID-19 patients show immune dysregulation and more severe symptoms.

## 1. Introduction

Since the end of 2019, the COVID-19 outbreak has spread almost all over the world at an exponential rate. Although the presentation of vaccines in early 2021 may decrease the rate of studies in COVID-19 treatments, new variants of SARS-CoV-2 with a high potency of escaping conventional vaccines motivate us to search for risk factors involved in the occurrence of severe cases of the disease.

The clinical manifestations of COVID-19 vary from asymptomatic and mild to severe forms. Some evidence has been estimated that the severe form of COVID-19 threatens 1.7 billion people (1 in 5) around the world due to at least one risk factor [1]. The severe form progresses rapidly to pneumonia and acute respiratory distress syndrome (ARDS) and even death. Several factors have been proposed as risk factors for the disease severity including comorbidities (i.e., diabetes, obesity, and cardiovascular diseases), age, sex, ethnicity, and genetic predispositions [2].

The main targets of SARS-CoV-2 for cell entry are angiotensin-converting enzyme 2 (ACE2) and some serine proteases such as transmembrane serine protease 2 (TMPRSS2). After virus entrance, intracellular antiviral mechanisms lead to the secretion of interferons (IFNs) following the recognition of virus components by host pattern recognition receptors (PRRs), which trigger an inflammatory response by activation of nuclear factor kappa B (NF*κ*B) [3].

Inflammation is the main part of the host response to any pathogen; however, patients with COVID-19, particularly in the severe form of the disease, catastrophically show signs of acute hyperinflammation and produce systemic markers of inflammation such as CRP, high levels of white blood cells, and proinflammatory cytokines [4]. The significant positive effect of dexamethasone on hospitalized patients in the RECOVERY study confirms the role of hyperinflammation in the pathogenesis of severe form of COVID-19 [5].

One of the main findings of severe COVID-19 is the increase in neutrophil (neutrophilia) and decrease in lymphocyte (lymphopenia) counts (high NLR). These symptoms reveal a dysregulated form of the immune response as a result of highly activated innate immunity [6]. In particular, high levels of neutrophils attack infected tissues and by releasing toxic substances such as proteases, neutrophil extracellular traps (NET), and reactive oxygen species (ROS) contribute to tissue injury [7]. NLR is used as a prognostic marker in critically ill patients like sepsis. This may be a criterion of immune response dysregulation and COVID-19 outcomes [6].

If the role of dysregulated immune response and increase of NLR in the pathogenesis of severe COVID-19 illness are accepted in an algorithmic approach, it is plausible to consider the natural immune-modulatory mechanisms and probable impairment of these mechanisms in some people.

AAT is first recognized as an antiprotease that mainly releases from the liver and inhibits proteases derived from activated neutrophils, particularly those infiltrated in the lungs. AAT is encoded by the *SERPINA-1* gene and expressed as a 51 kD glycoprotein [8]. In addition to the role of AAT in the neutralization of proteases and ROS produced by neutrophils, recent studies have suggested that AAT plays anti-inflammatory and antiviral roles in humans [9]. Some other proteases involved in SARS-CoV-2 cell entries, such as TMPRSS2, seem neutralized by this antiprotease [10]. AAT with immunomodulatory effects has no immunosuppressive result and no impairment of normal immune response. Several mutations in the *SERPINA-1* gene lead to hepatic protein synthesis defection and AAT deficiency (AATD) [8]. Individuals with homozygote mutation suffer from respiratory and hepatic diseases, while the heterozygotes are often asymptomatic. The heterozygote forms of AATD may have been underestimated in different geographical areas; this may be the cause of the susceptibility of some patients to the serious types of COVID-19 [11]. The main purpose of this study was to analyze serum AAT levels and explore the association of NLR and AAT with the severity of COVID-19 in hospitalized patients.

## 2. Methods

### 2.1. Study Design and Participants

**Figure 1:**
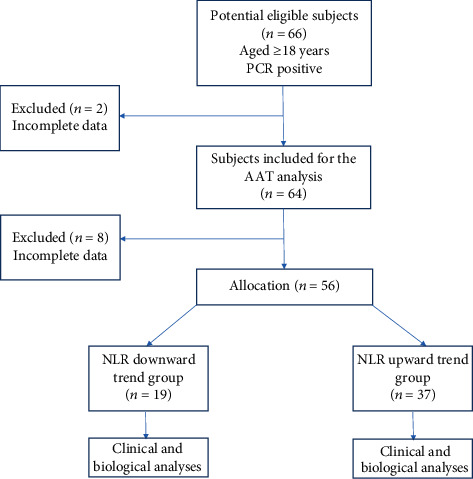
Flow chart of case enrolment in this retrospective observational cohort study between July 2020 and November 2020. Abbreviations: AAT: alpha-1 antitrypsin; NLR: neutrophil-lymphocyte ratio; PCR: polymerase chain reaction.

This retrospective observational cohort study was performed on 64 consecutive hospitalized COVID-19 patients who were admitted to the hospitals affiliated to Shahid Beheshti University of Medical Sciences from July 2020 to November 2020 (Figure 1). Patients (aged ≥ 18 years) included in the study were diagnosed with COVID-19 based on diagnostic criteria developed by the World Health Organization for COVID-19 (detection of virus RNA through PCR assay from nasal and pharyngeal swab specimens) [12]. All patients were diagnosed as severely ill with fever, cough, fatigue, muscle and joint pain, shortness of breath, and ARDS. Also, they were mostly admitted to the intensive care unit (ICU). Written informed consent forms were received from all participants before recruitment. All eligible patients were followed up until attaining outcome measures, passing away, or discharging from the hospitals. The study was approved by the ethics committee at the National Nutrition and Food Technology Research Institute of Shahid Beheshti University of Medical Sciences (code of ethics committee: IR.SBMU.NNFTRI.REC.1399.059).

### 2.2. Participants' Characteristics and Data Collection

Clinical and biological characteristics of the patients were obtained from the hospital's electronic medical records. Data were recorded by data entry teams into a computerized database and reviewed by two experienced clinicians. Recorded comorbidities included any type of cancer, type 2 diabetes mellitus, and heart diseases (heart failure, coronary artery disease, and pulmonary hypertension).

### 2.3. Laboratory Data

AAT was determined by an immunoturbidimetric assay using a Roche COBAS INTEGRA 400 plus analyzer (Rotkreuz, Switzerland) with 20 mg/dl sensitivity. Red blood cells (RBC), platelets, neutrophil, and lymphocyte counts and their percentages were analyzed automatically by a blood cell counter (Beckman Coulter, Miami, FL, USA). CRP, random blood sugar (BS), SGOT, and SGPT were measured by using the diagnostic kits from Pars Azmoon Company (Pars Azmoon Co., Tehran, Iran) and an autoanalyzer Selectra ProXL (Vital Scientific, Spankeren, The Netherlands). The kits used in this study had a sensitivity of 5 mg/dl for BS, 2 IU/l for SGOT, and 4 IU/l for SGPT. Measurement of ESR was made using the Westergren method and ESR tube according to standard procedure. Troponin concentration was examined by an enzyme-linked immunoabsorbent assay (ELISA) kit (Monobind Inc., Lake Forest, CA, USA). The NLR value was calculated by dividing the neutrophil counts by the lymphocyte counts. O2sat was measured directly by an ABL90 FLEX PLUS blood gas analyzer (Copenhagen, Denmark). Blood samples were taken at admission and during hospitalization. Only the first and last test results of neutrophil and lymphocyte counts, CRP, ESR, and NLR were recorded.

### 2.4. Clinical Outcomes

The primary aim of this study was to examine whether the serum level of AAT reduces in the COVID-19 patients. Moreover, the study focused on two patient subtypes: cases with NLR decreasing trend and those with NLR increasing trend during the study. Differences in patients' clinical and laboratory characteristics were evaluated between the two subtypes.

### 2.5. Statistical Analyses

Statistical analyses were performed by using SPSS software, version 20. A two-tailed *p* value < 0.05 was considered statistically significant. Continuous variables were presented as means ± SD or medians with interquartile ranges (IQRs), based on the normal or skewed distribution. Categorical variables were presented as number (%). The independent-samples *t*-test for normally distributed variables and the Mann-Whitney *U* test for nonnormally distributed data were used. For categorical variables, the chi-square (*χ*^2^) test and Fisher's exact or Fisher-Freeman-Halton test were applied [13].

## 3. Results

This study includes 64 severe COVID-19 patients, who were mostly hospitalized in the ICU. However, few cases were excluded due to some missing parameters, later on (Figure 1). They were admitted to the hospitals with common symptoms of severe COVID-19 including fever, dyspnea, low oxygen saturation, and cough which had been started at least seven days prior to the hospitalization. The demographic, clinical, and laboratory characteristics are shown in Table 1. The mean (±SD) age of patients was 58.66 ± 14.97 years. There were 66% percent males and 34% females. Comorbidities were reported only in 46 patients: heart diseases (24%), diabetes mellitus (21.7%), and cancer (11%).

The participants were subdivided into two groups according to increased or decreased neutrophil to lymphocyte ratio during hospitalization. The overall mortality rate was 41%, significantly different between NLR upward (51%) and downward (21%) groups (*p* < 0.05). Comorbidities were not significantly different between NLR downward and upward trend groups.

Expectedly, most patients were admitted with high levels of CRP caused by the inflammatory disease. Following the first week of hospitalization, some of the patients showed a decrease in CRP levels, while the value continued to rise in the rest. Also, there was no significant difference between CRP alterations in NLR upward and downward trend groups. ESR changes—as the inflammation marker—were similar to CRP. The baseline values were higher in most patients, in some ESR levels decreased during hospitalization, and in others increased. The NLR did not associate with ESR alterations.

Troponin was another marker that increased in most patients. The serum level of this cardiac biomarker was significantly higher in the NLR upward trend group (*p* < 0.05). Nonfasting blood sugar in severe COVID-19 patients was higher than the normal range (less than 140 mg/dl), though the glucose levels were not significantly different between the NLR groups. Liver enzymes, SGOT, and SGPT also encountered moderate changes in many patients compared to the normal range. However, this increase was not different between NLR upward and downward trend groups. RBC and PLT values were also not significant between the two groups. Moreover, O2sat was slightly higher in the NLR downward trend group, although the difference between the two groups was not significant.

**Figure 2:**
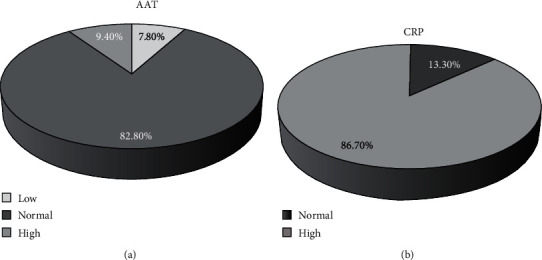
Pie charts showing percentages of AAT and CRP in patients included in this study. (a) Percent of patients in low (≤90 mg/dl), normal (90-200 mg/dl), and high (≥200 mg/dl) ranges of AAT (*n* = 64). (b) Percent of patients in normal (<6 mg/l) and high (≥6 mg/l) ranges of CRP (*n* = 60). Abbreviations: AAT: alpha-1 antitrypsin; CRP: C-reactive protein.

Finally, the current study results showed that 82.80% of patients had normal AAT levels. The percentages of individuals with low and high AAT levels were 7.80% and 9.40%, respectively (Figure 2(a)). Moreover, the high and low values of CRP were 86.70% and 13.30% (Figure 2(b)), indicating that in spite of having serum AAT levels mostly in the low and normal ranges, the majority of study subjects had high CRP values, at the time of hospitalization.

## 4. Discussion

The results of the present study showed that severe COVID-19 patients had serum AAT levels mostly in the normal range at the time of admission, despite high baseline levels of CRP and ESR. The patients who showed an increasing NLR trend during hospital attendance had a higher mortality rate and slightly lower, but not significant, serum AAT levels than patients with decreasing NLR trend. Participants with the upward NLR trend had also higher serum troponin levels. The baseline levels of CRP, ESR, and their alterations during hospitalization were not significantly different between increasing and decreasing NLR trend groups.

We observed 41% mortality in patients diagnosed as severe COVID-19. In a Mexico City study, 31% of patients admitted to hospital had died as severe COVID-19 [14]. This discrepancy may be due to the difference in sample size or comorbidities among patients who participated in these two studies.

AAT is an acute-phase plasma protein that increases by 75 to 100% during inflammation accompanied by other acute-phase proteins such as CRP or ESR. Compared to baseline levels, the inflammation-induced elevation of AAT is observed in the individuals with homozygote and heterozygote forms of AATD. Therefore, normal levels of AAT in AATD may be due to the inflammation that induces elevation of lower baseline. It has been shown that CRP is not influenced by AATD; therefore, AAT should be considered in correlation with CRP to better estimate AAT response in different genotypes of AAT during normal and inflammatory states [15]. CRP levels were elevated in our participants; however, the levels of AAT remained in the normal range. The normal or less than normal levels of AAT in COVID-19 patients show an inadequate AAT response in severe COVID-19 patients. With reference to a study conducted by McElvaney et al., although the AAT levels increased in COVID-19 critically ill patients, its acute-phase response was not sufficient because of failing to keep pace with interleukin-6 (IL-6). Therefore, COVID-19 patients admitted to the ICU in their study had a significantly higher IL-6 compared to the severe community-acquired pneumonia (COPD) ICU patients, and no difference in serum circulating levels of AAT was observed between them [15]. According to baseline levels of CRP and ESR, it has been expected that the AAT levels should be higher. One proposed explanation for lower AAT response to COVID-19 severe patients is the higher prevalence of AATD among severe COVID-19 patients. According to Johns Hopkins University, Spain, Italy, United Kingdom, and France have the highest prevalence of *PI*∗*ZZ*, *PI*∗*SZ*, and *PI*∗*MZ* genotypes compared to other parts of Europe and the world. Interestingly, the mortality rate of these countries from severe COVID-19 cases was the highest among other countries [11, 16]. Vianello and Braccioni emphasized the paradigm by showing that most of the registered cases of COVID-19 were in northern Italy particularly in the Lombardia region. The authors proposed that the high rate of COVID-19 in the north of Italy may be consistent with the highest prevalence of AATD in northern regions of Italy [17]. Besides genetic variations in AAT expression and production, there is some evidence showing that AAT production may decrease under certain conditions in an acquired manner. For instance, it has been shown that AAT and 25(OH) vitamin D are lower in type 2 diabetic patients [18]. Mounting evidence indicates that both diabetes and vitamin D deficiency are potential risk factors in COVID-19 [19, 20].

The elevation of NLR is due to increased neutrophils or decreased lymphocyte counts. NLR has potential utility in clinical applications for the prognosis of inflammatory diseases including ischemic stroke [21], cerebral hemorrhage [22], major cardiac events [23], chronic obstructive pulmonary disease [24, 25], and some types of cancers [26–28]. Association of NLR with the severity of COVID-19 illness has been subjected to considerable attention from researchers worldwide [6, 29–31]. Neutrophils, as the most abundant leukocyte, are heterogeneous subpopulation of immune cells. Activated neutrophils release proteases, ROS, and NETs to fight pathogens. Uncontrolled release of these factors has great potential to damage infected tissues [7]. It has been shown that SARS-CoV-2 infection promotes neutrophil activation and count. Wang et al. showed that higher levels of neutrophils are accompanied by lung damage in severe COVID-19 [32]. The present study also showed that severe cases of COVID-19 with an increasing NLR had higher mortality compared to patients with decreasing trend of NLR. In addition, NLR upward trends had lower serum levels of AAT, but nonsignificant. AAT is one of the major regulators of neutrophil hyperactivation through both neutralizations of their serine proteases and immunomodulatory function. For instance, AAT inhibits superoxide and inflammatory cytokine production which are induced by activated neutrophils [33, 34].

The recent evidence indicates that SARS-CoV-2 had an adverse effect on the cardiovascular system, and many patients treated for COVID-19 may face thrombosis and heart attack, a few months after treatment [35]. We observed that in participants with increasing NLR tendency, troponin levels were higher than participants with decreasing NLR trends. It appears that patients with severe COVID-19 have higher troponin levels [36]. Some recent data revealed that elevation of troponin is accompanied by higher hospital mortality [37].

In conclusion, the results of the present study demonstrate that severe COVID-19 patients may release inadequate amount of AAT proteins in the blood circulation and thus encounter neutrophilia and lymphopenia. Therefore, AAT response may be considered as a new mechanism by which some COVID-19 patients show dysregulated inflammation and more severe symptoms. Since AAT-rich plasma is administered to individuals with AATD and some other conditions such as diabetes type 1 and influenza, it is plausible to consider it as a new treatment for severe COVID-19 patients.

## Figures and Tables

**Table 1:** Clinical and laboratory analyses of the patients and differences in patients' characteristics between NLR downward trend and NLR upward trend groups.

Variables	Total	NLR downward trend	NLR upward trend^#^	*p* value^∗^
*N*	56	19	37	
Gender				0.790
Male, *n* (%)	37 (66.1)	13 (68.4)	24 (64.9)	
Age (year, mean ± SD)	58.66 ± 14.97	56.53 ± 16.19	59.76 ± 14.42	0.450
Mortality				0.029
Yes, *n* (%)	23 (41.1)	4 (21.1)	19 (51.4)	
No, *n* (%)	33 (58.9)	15 (78.9)	18 (48.6)	
Comorbidities, *n* (%) (*n* = 46)				0.121
No, *n* (%)	20 (43.5)	8 (57.1)	12 (37.5)	
Cancer, *n* (%)	5 (10.9)	3 (21.4)	2 (6.2)	
Diabetes mellitus, *n* (%)	10 (21.7)	2 (14.3)	8 (25)	
Heart diseases, *n* (%)	11 (23.9)	1 (7.1)	10 (31.2)	
AAT (mg/dl, mean ± SD)	132.65 ± 43.44	136.68 ± 51.63	130.53 ± 39.07	0.622
CRP (*n* = 35)				0.903
Downward trend, *n* (%)	18 (51.4)	6 (50)	12 (52.2)	
Upward trend, *n* (%)	17 (48.6)	6 (50)	11 (47.8)	
ESR (*n* = 34)				0.715
Downward trend, *n* (%)	16 (47.1)	4 (40)	12 (50)	
Upward trend, *n* (%)	18 (52.9)	6 (60)	12 (50)	
RBC (10^6^/*μ*l, mean ± SD)	4.19 ± 0.74	4.11 ± 0.98	4.24 ± 0.63	0.622
PLT(10^3^/*μ*l, mean ± SD)	192.80 ± 81.85	211.11 ± 70.21	178.49 ± 88.12	0.167
O2sat (%, mean ± SD)	76.28 ± 21.68	83.28 ± 16.59	73.81 ± 23.13	0.369
BS (mg/dl, mean ± SD)	188.54 ± 89.63	183.5 ± 74.97	190.56 ± 96.21	0.837
SGOT (U/l, median (IQR))	57.36 ± 47.79	53.00 (46)	42.00 (22.25)	0.811
SGPT (U/l, mean ± SD)	51.40 ± 30.32	56.20 ± 21.88	49.16 ± 33.64	0.464
Troponin (ng/ml, mean ± SD)	0.03 ± 0.02	0.02 ± 0.02	0.04 ± 0.02	0.011

Abbreviations: OR: odds ratio; NLR: neutrophil-lymphocyte ratio; AAT: alpha-1 antitrypsin; CRP: C-reactive protein; ESR: erythrocyte sedimentation rate; PLT: platelet; RBC: red blood cells; O2sat: arterial oxygen saturation; BS: random blood sugar; SGOT: serum glutamic oxaloacetic transaminase; SGPT: serum glutamic pyruvic transaminase. Normal range: AAT: 90-200 mg/dl; CRP: >10 mg/l; RBC: 3.8-5.8 10^6^/*μ*l; PLT: 150-450 10^3^/*μ*l; O2sat: 95-99%; BS: up to 140 mg/dl; SGOT: up to 41 U/l; SGPT: up to 41 U/l; troponin: <0.04 ng/ml. Note: data are shown as mean ± SD, median with interquartile range (IQR), and number (%). ^∗^Independent-samples *t*-test or Mann-Whitney was used for continuous variables with the normal and nonnormal distribution, respectively. The chi-square test and Fisher's exact or Fisher-Freeman-Halton test were used for categorical variables. ^#^NLR upward trend group consisted of individuals with having an upward NLR trend along with those with no NLR change during the study.

## Data Availability

All data are achieved from SBMU hospitals and deposited in our department for any further applications and publicity.
